# Ambient light level varies with different locations and environmental conditions: Potential to impact myopia

**DOI:** 10.1371/journal.pone.0254027

**Published:** 2021-07-07

**Authors:** Shashank Kishore Bhandary, Rohit Dhakal, Vishwa Sanghavi, Pavan Kumar Verkicharla

**Affiliations:** Prof. Brien Holden Eye Research Centre, Myopia Research Lab, Brien Holden Institute of Optometry and Vision Sciences, L V Prasad Eye Institute, Hyderabad, India; National Taiwan University Hospital, TAIWAN

## Abstract

**Purpose:**

Considering that time spent outdoors is protective for myopia, we investigated how ambient light levels reaching the eye varies across 9 outdoor and 4 indoor locations in 5 different environmental conditions.

**Methods:**

Illuminance (lux) was recorded using a lux meter under conditions of weather (sunny/cloudy), time of a day (7:00,10:00,13:00, and 16:00 hours), seasons (summer/winter), and sun protection (hat and cap) in outdoor and indoor locations. Nine outdoor locations were “open playground”, “under a translucent artificial-shade”, “under a porch facing east”, “under a porch facing south”, “under a big tree”, “between three buildings”, “within 4 buildings”, and “canopy”. As a ninth outdoor location, “Under a glass bowl” in the outdoor location was used as a simulation for “glass classroom model” and measurement was taken at the floor level only to determine in overall the illuminance conditions with glass covered on all sides. The 4 indoor locations included “room with multiple large windows”, “room with combination light source”, “room with multiple artificial lights”, and “room with single artificial light”.

**Results:**

The overall median illuminance level (median; Q1-Q3) recorded in 9 outdoor locations was 8 times higher than that of all indoor locations (1175;197–5400 lux vs. 179;50–333 lux). Highest illuminance in outdoor locations was recorded in “open playground” (9300;4100–16825 lux), followed by “under a translucent artificial shade (8180;4200–13300 lux) and the lowest in “within 4 buildings” (11;6–20 lux). Illuminance under ‘Canopy’, ‘between three buildings’ and ‘within four buildings’ was similar to that of indoor locations (<1000 lux). Time of the day, weather, season, sensor position and using sun protection did not alter illuminance to change from high to low level (>1000 to <1000 lux). Among indoor locations, illuminance in “room with multiple large windows” crossed 1000 lux at a specific time points on both sunny and cloudy days.

**Conclusions:**

Illuminance levels in outdoors and indoors varied with location type, but not with other conditions. Given the variation in illuminance in different locations, and the impact it may have on myopia control, appropriate detailed recommendations seems necessary while suggesting time outdoors as an anti-myopia strategy to ensure desired outcomes.

## Introduction

The increasing prevalence of myopia and its associated complications due to ocular stretching necessitates appropriate intervention for myopia control [1–3]. Based on several studies, time spent outdoors is considered protective for myopia [4–10]. Recently published systematic review and meta-analyses indicated time outdoors to be protective for myopia or to delay the onset of myopia [11–13]. While Sherwin et al. [12] reported an additional hour per week of outdoor activities can reduce odds of becoming myopic by 2%, Ho et al. [11] suggested 120 minutes of daily outdoor exposure during school hours as the most effective intervention in controlling myopia. Studies that quantified the light exposure pattern by estimating illuminance using a light tracker reported that myopic children spent most of their time in an indoor environment with illuminance level <1000 lux [4, 5, 14–16]. A recent study reported reduction in odds of developing myopia when exposed to an illuminance level of >3000 lux every day [14].

Children at school mostly spend their time in an indoor environment such as classroom and relatively less time in an outdoor environment such as playground [17]. There can be several variations in an indoor and outdoor environment. For example, a playground can have an open-top or will be covered by a big tree shade or a roof where children play. Similarly, a typical indoor environment such as a classroom can be with no window, a single window, or multiple windows, along with a single or multiple light sources. School corridors can be open on one side or completely closed from both sides and so are the other places where children spend their time [8]. There are several other conditions such as weather (sunny or cloudy), geographical locations, elevations, seasons (summer or winter) and time of the day (morning, noon, afternoon or evening) that might alter the illuminance level in different locations. The direction of the sunlight towards sight of children while playing can also affect the illuminance levels reaching the eye level [18]. For example, illuminance levels can be higher when facing the sun than that recorded opposite to the sun. The trend of using hat/cap while going outdoors to protect skin burn and other ultraviolet (UV) related damage to eye can also impact the amount of illumination reaching the eye.

Dharani et al. [16] performed a pilot study using a pendant type light meter and reported high illuminance levels in an outdoor location on a bright sunny day (278919–30311 lux) compared to that of a dark cloudy day (3896–7559 lux) and least in indoors (<1000 lux). Lanca et al. [19] reported that use of sunglasses/hat still provided illuminance (at the eye level) 11–43 times higher than that of indoors. The outdoor locations investigated by Lanca et al. [19] were limited to an open field environment, under the shade of tree and street, and indoor locations were limited to a room with the fluorescent lamp and open window, and a room with cool light-emitting diode (LED) without window.

Considering that multiple factors can affect light levels reaching the eye, understanding illuminance levels in different locations, where children spend most of their time would be beneficial in making appropriate recommendations while suggesting time outdoors as an anti-myopia strategy. To the best of our knowledge, quantification of illuminance levels in different outdoor and indoor locations where school-going children spend most of their time has not been studied extensively. This study investigated how ambient illuminance levels reaching the eye vary with weather (sunny/cloudy), time of a day, sensor source position (eye position in relation to source), sun protection with hat/cap and seasons (summer/winter) in 9 outdoor and 4 indoor locations.

## Methods

This is an experimental study conducted in the first week of June and November in the year 2019 at Hyderabad, which is the capital city of Telangana state that is in southern part of India. Illuminance levels in ‘lux’ were obtained using a factory-calibrated digital lux meter (Sinometer LX-1330B with 0 to 200000 lux output range) by a single examiner in nine outdoor and four indoor locations. To maintain accuracy of recording illuminance levels at each location, the display screen was auto zeroed by closing the cap of the sensor as recommended in the manual provided by the lux meter manufacturer [20]. The measurements of illuminance levels were obtained by keeping the sensor of a lux meter at the level of examiner’s eye (5.6 feet above the ground) to obtain a closer value of illuminance level that enters the eye. Whereas, illuminance levels obtained in a glass bowl location were recorded with the sensor of the lux meter facing upwards. Three consecutive readings were noted for each measurement condition in all the locations and average of these readings was used for further analysis.

### Locations used in the study

**Fig 1:**
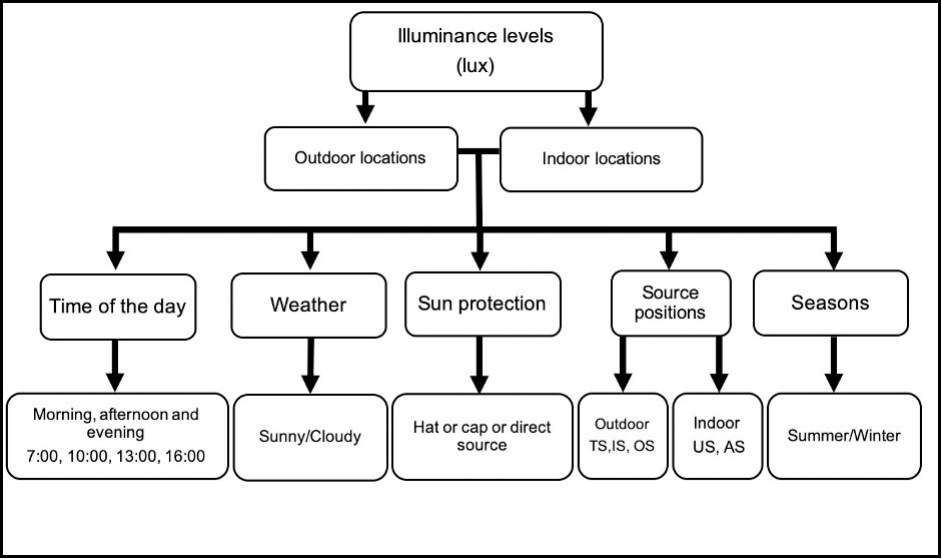
Overview of measured description in this study under different conditions with their illuminance levels recorded. Sensor source positions: TS—Towards the source, IS—Intermediate from the source, OS—Opposite to the source, US—under the source and AS—away from the source.

The description of all locations is given in Table 1 along with the pictorial representation. Different measurement conditions influencing these locations are shown in Fig 1. Nine outdoor locations were “open playground”, “under a translucent artificial-shade”, “under a porch facing east”, “under a porch facing south”, “under a big tree”, “between three buildings”, “within 4 buildings”, and “canopy”. As a ninth outdoor location, “Under a glass bowl” in the outdoor location was used as a simulation for “glass classroom model” and measurement was taken at the floor level only to determine in overall the illuminance conditions with glass covered on all sides. Likewise, 4 indoor locations were a “room with multiple large windows”, “room with combination light source”, “room with multiple artificial lights”, and “room with single artificial light”.

**Table 1:** List of nine outdoor and four indoor locations with their description. Pictorial presentation of these locations are available in S1 File.

Locations		Description
	**Outdoors**	
**1**	Open playground	Big open space used for playing.
**2**	Under big tree	One big tree present in a playground and illuminance was recorded under the shade of a tree.
**3**	Under a translucent artificial shade	A translucent sloping roof covering a small area in an open space. Illuminance measurements were taken under the roof.
**4**	Canopy	An area covered by many heavy tree crowns.
**5**	Under a glass bowl	A glass bowl with a transparent base and translucent sides placed upside down in the open space with a lux-meter sensor inside the bowl
**6**	Under a porch facing east	A porch is a covered shelter placed in front of a house or a building. The position of this porch is facing the cardinal east direction.
**7**	Under a porch facing south	The position of the porch is facing the cardinal south direction.
**8**	Between 3 buildings	A walkaway connected to the road enclosed by three tall buildings on three sides. Usually found in multi clustered apartments.
**9**	Within 4 buildings	An area covered by buildings on all four sides with open sky. Usually found in multi clustered apartments.
	**Indoors**	
**1**	Room with multiple large windows	A large room (dimensions in meters: length X breadth X height—11.9 X 22.5 X 9.6) with windows covering until two floors facing cardinal east position with exposure to natural light.
**2**	Room with a combination light source	A room (dimensions in meters: 5.9 X 5.9 X 2.5) consisting of windows allowing natural light to enter the room and overhead LED lamps from a ceiling, example library.
**3**	Room with multiple artificial lights	The room (dimensions in meters: 9.9 X 9.3 X 3.2) consisted of multiple LED white lamps aligned sequentially on the ceiling and no natural source of light.
**4**	Room with single artificial light	A closed room (dimensions in meters: 5.8 X 2.9 X 2.8) consisting of a single artificial fluorescent lamp which illuminates the whole room

### Time of the day, seasons, and weather

The illuminance level was recorded across four different time points in a day (7:00–8:00, 10:00–11:00, 13:00–14:00 and 16:00–17:00 hours) each on two sunny and two cloudy days. Measurements were obtained in both winter (November 2019) and summer (June 2019) seasons to compare the variance of illuminance level between the two most observed seasons in major parts of the country. The forecast of the day (as sunny or cloudy) was recorded from the default weather application on an android smartphone (AccuWeather application, https://www.accuweather.com/en/in/hyderabad/202190/weather-forecast/202190). Out of 9 outdoor locations, illuminance levels was recorded only in 3 outdoor locations during the summer season (“open playground, “under a glass bowl”, and “under a translucent artificial shade”). Indoor locations were mostly used in summer season as a comparison for classroom levels so, the outdoor locations were limited in illuminance measurements.

### Different positions of sensor relative to the source (sensor-source position)

The measurements in outdoor locations were obtained by positioning the sensor of the lux meter in three different directions relative to the source of light, i.e. i) facing towards the source—TS, ii) facing opposite to the source—OS, and iii) facing intermediate to the source—IS which is facing 90 degrees midway from both TS and OS. Likewise, for indoor locations, measurements were obtained for two different positions of lux meter sensor relative to the location of artificial light source i.e. i) under the source- US (directly under the source of light with the sensor facing towards the ceiling), and ii) away from the source- AS (depending on the size of indoor location and placement of sources of light on the ceiling, measurements were obtained at a distance of 1–2 meters away).

### Measurements using sun protection

To investigate the influence of using sun protection with hat or cap white in colour on the illuminance level reaching to an eye-level, all the above measurements were obtained under three conditions in the outdoor locations: i) direct exposure to sunlight, ii) after wearing a round brimmed white colour hat, and iii) after wearing a cap.

### Statistical analysis

The data were recorded and analysed using MS-Excel 2016 (Microsoft Corporation, USA) and was presented as median; Inter Quartile Range (IQR) for different outdoor and indoor locations. The term “overall” was defined as the illuminance levels recorded in all locations depending on different conditions (time of the day, weather, seasons, relative sensor-source position and using sun protection with hat or cap).

## Results

### Illuminance levels in outdoor and indoor locations

**Fig 2:**
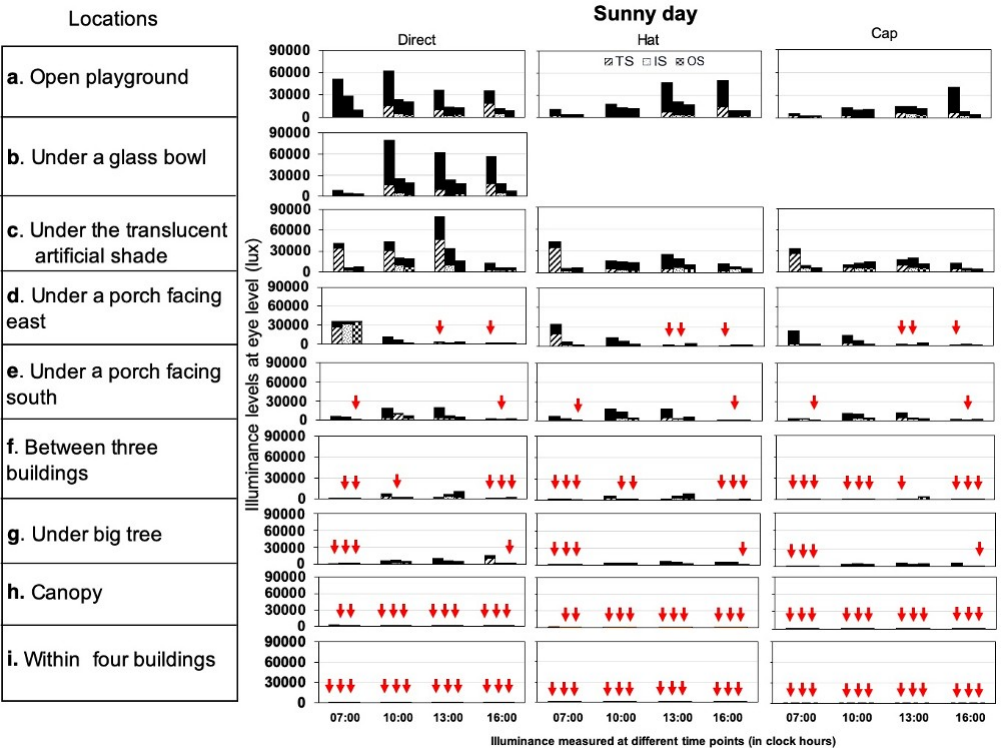
Illuminance levels recorded under direct source, hat and with a cap on two sunny days. TS, IS and OS sensor source positions recorded in four different time points of a day (07:00–08:00, 10:00–11:00, 13:00–14:00 and 16:00–17:00 hours). The dark shaded area on the top of each bar represents the difference in illuminance levels obtained on two different sunny days. The downward-facing arrows in red colour represent illuminance levels <1000 lux.

**Fig 3:**
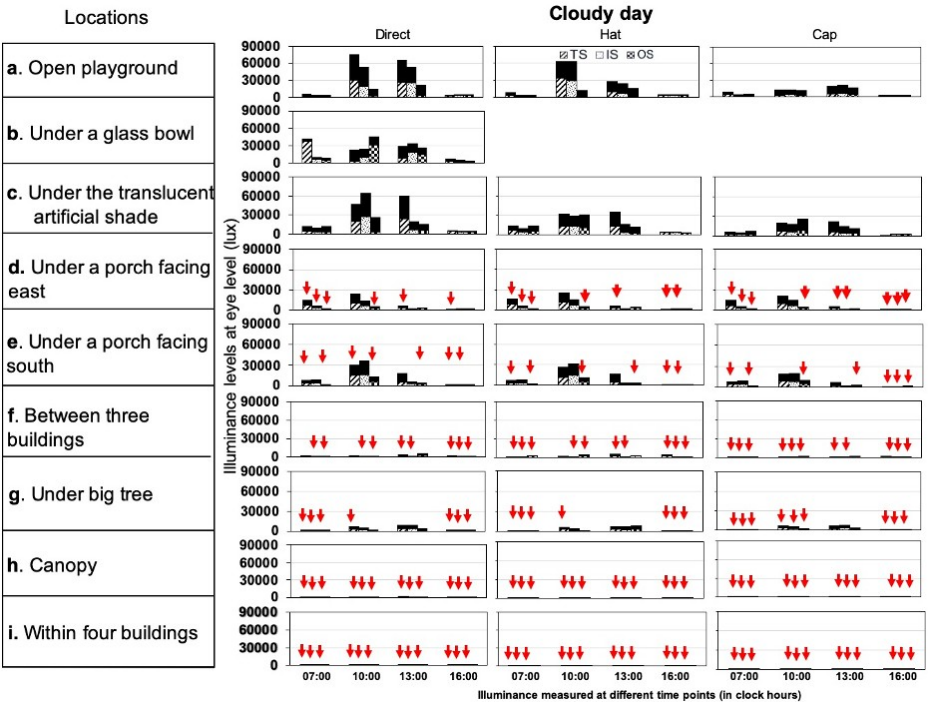
Illuminance levels recorded under direct source, hat and with a cap on two cloudy days. TS, IS and OS sensor source positions recorded in four different time points of a day (07:00–08:00, 10:00–11:00, 13:00–14:00 and 16:00–17:00 hours). The dark shaded area on the top of each bar represents the difference in illuminance levels obtained on two different sunny days. The downward-facing arrows in red colour represent illuminance levels <1000 lux.

The overall median illuminance level recorded across all outdoor locations was 8 times higher than that of the indoor locations (1175; 197–5400 lux vs. 179; 50–333 lux). Overall, the highest median illuminance levels under all the conditions in outdoor locations was recorded in an “open playground” (median: 9300; 4100–16825; maximum: 93500 lux), followed by “under a translucent artificial shade (median: 8180; 4200–13300; maximum: 79900 lux) and the lowest in “within 4 buildings” (median: 11; 6–20; maximum: 102 lux). Of all these included outdoor locations, only two outdoor locations, i.e. “open playground”, and “under a translucent artificial shade” recorded median high illuminance levels ≥ 1000 lux irrespective of different measurement conditions previously stated in Table 2. Three outdoor locations namely, “under a porch facing south”, “under a porch facing east” and “under a big tree although recorded overall median high illuminance levels >1000 lux, even in conditions such as time of the day in sunny or cloudy weather. These locations also showed low illuminance levels (<1000 lux) with different sensor source positions and thus lacked in maintaining high illuminance levels consistently (Figs 2 and 3). The median illuminance for “under a porch facing east or south” and under a big tree varied by 600 lux with overall illuminance levels ranging from 1500 to 2100 lux in these 3 locations. The “under a glass bowl” location which was used as a simulation for “glass classroom model” in outdoor location recorded illuminance levels (median: 13300; 4075–20550; maximum: 80200) similar to the range obtained in an “open playground”. Outdoor locations such as “between three buildings”, “canopy” and “within four buildings” showed overall low median illuminance levels of <1000 lux in all conditions (Figs 2 and 3).

**Table 2:** Overall and direct median (IQR) illuminance levels with range recorded in different outdoor and indoor locations (irrespective of the time of the day, sunny or cloudy weather, relative sensor source positions, use of sun protection, and summer and winter season).

S. No	Location	Overall illuminance values (lux)		Direct illuminance values (lux)	
		Median (IQR)	Range	Median (IQR)	Range
	**Outdoor locations**				
**1**	Open playground	9300 (4100–16825)	440–93500	14350 (7100–27375)	1120–93500
**2**	Under a glass bowl			13300 (4075–20550)	910–80200
**3**	Under the translucent artificial shade	8180 (4200–13300)	415–79900	9600 (5760–20000)	578–79900
**4**	Under a porch facing south	2080 (865–5200)	122–20500	2200 (893–5275)	212–20500
**5**	Under a porch facing east	1400 (535–3003)	30–36400	1685 (580–3033)	40–36400
**6**	Under big tree	1580 (548–3400)	75–15000	1700 (675–3425)	96–15000
**7**	Between 3 buildings	395 (168–1033)	36–9080	500 (200–500)	56–9080
**8**	Canopy	162 (99–310)	7–2600	178 (99–310)	21–2600
**9**	Within 4 buildings	11 (6–20)	1–102	17 (8–27)	4–102
	**Indoor locations**				
**1**	Room with multiple large windows	430 (173–2625)	30–28500	2650 (817–5615)	350–28500
**2**	Room with multiple artificial lights	209 (190–300)	160–510	290 (234–337)	200–510
**3**	Room with a combination light source	91 (72–225)	40–646	240 (89–397)	40–646
**4**	Room with single artificial light	14 (12–16)	9–116	14 (11–32)	9–116

Note: “Overall” includes data with different sun protection, time points, weather, and source position in outdoor locations; for indoors this includes data with different time points, weather, and source position. “Direct” includes without sun protection.

Indoor locations showed an overall low median illuminance levels (i.e. <1000 lux) irrespective of different measurement conditions. However, the overall median illuminance levels in a “room with multiple large windows” crossed 1000 lux at specific time points such as 10:00 and 13:00 hrs. on both sunny and cloudy weather conditions and showed the highest illuminance among all indoor locations (median: 179; 50–333; maximum: 28500 lux).

### Illuminance levels at different time points of a day

**Fig 4:**
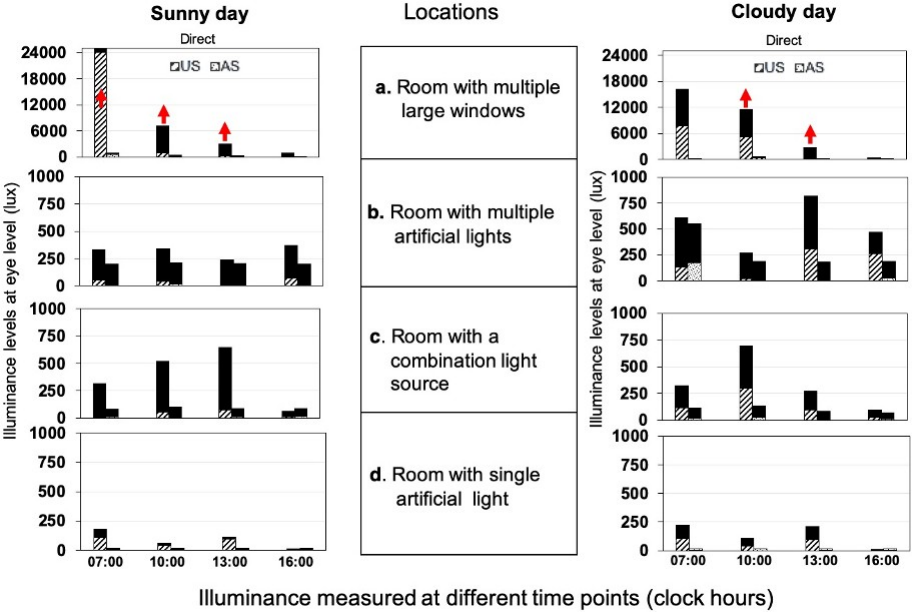
Illuminance levels recorded in four indoor locations on two sunny and cloudy days. US and AS sensor source positions recorded in four different time points in a day (07:00–08:00, 10:00–11:00, 13:00–14:00 and 16:00–17:00 hours) in a sunny (left panel) and a cloudy day (right panel). The dark shaded area on the top of each bar represents the difference in illuminance level in two sunny and two cloudy days. The upward-facing arrows in red colour represent the illuminance level > 1000 lux.

The median illuminance levels of all the 9 outdoor locations together were >1000 lux during 07:00–08:00 hours (1300; 200–5250 lux), 10:00–11:00 hours (1400; 10–6000 lux), and 13:00–14:00 hours (1400; 200–5650 lux), but dropped to 1000 lux between 16:00–17:00 hours (1000; 167–4900 lux). In the indoor locations, the illuminance level in “a room with multiple large windows” and “a room with combination light source” changed with the time in a day. The illuminance in a “room with multiple large windows” gradually decreased from morning 07:00–08:00 hours to 16:00–17:00 hours, but always remained >1000 lux. Cloudy weather shifted the high illuminance levels from time point of 07:00 hrs to 10:00 hrs. Rooms with single artificial light did not show any change in illuminance levels with time in a day (Fig 4).

### Illuminance levels in sunny and cloudy days

The overall median (IQR) illuminance level recorded between the two sunny days or between two cloudy days across outdoor and indoor locations varied by 12–13% and 1–2%, respectively. The median illuminance level of all the outdoor locations together on a sunny day was 2.4 times higher than that of a cloudy day (1920; 300–7125 lux vs. 800; 150–3825 lux). The top three outdoor locations in Table 2 always showed illuminance levels >1000 lux in both sunny and cloudy days irrespective of different conditions. However, illuminance level under porch facing east, under porch facing south, and under a big tree although showed illuminance level >1000 lux in most of the measurement conditions during sunny days (Fig 2), dropped to <1000 lux on cloudy days (Fig 3). In the indoor locations, there was no effect of weather except for the illuminance level at multiple large windows which was < 1000 lux in the morning on a cloudy day (a sunny day at 07:00 hours—875 lux vs. 375 lux in a cloudy day). The median illuminance level in “open playground”, “under a glass bowl” and “under a translucent artificial shade” is 17 times higher than indoor locations in the sunny day (3,295 vs. 190 lux respectively) and 12 times higher in the cloudy days (1,150 vs. 98 lux respectively).

### Illuminance levels with sun protection, different sensor source positions and season

The median illuminance level of all the 9 outdoor locations was 1.4–1.7 times higher when the measurements were obtained directly without sun protection (1600; 308–6200 lux) than that recorded with hat (1185; 219–5200 lux) or cap (900; 160–4400 lux) (Figs 2 and 3). The sensor source position did not affect the overall illuminance level in outdoor locations (TS: 1400; 200–5795 lux; IS: 1240; 200–5400 lux; and OS: 1145; 192–5375 lux). In the indoor locations, the median illuminance level was similar in both US (180; 54–337 lux) and AS (169; 50–333 lux) sensor source positions.

**Fig 5:**
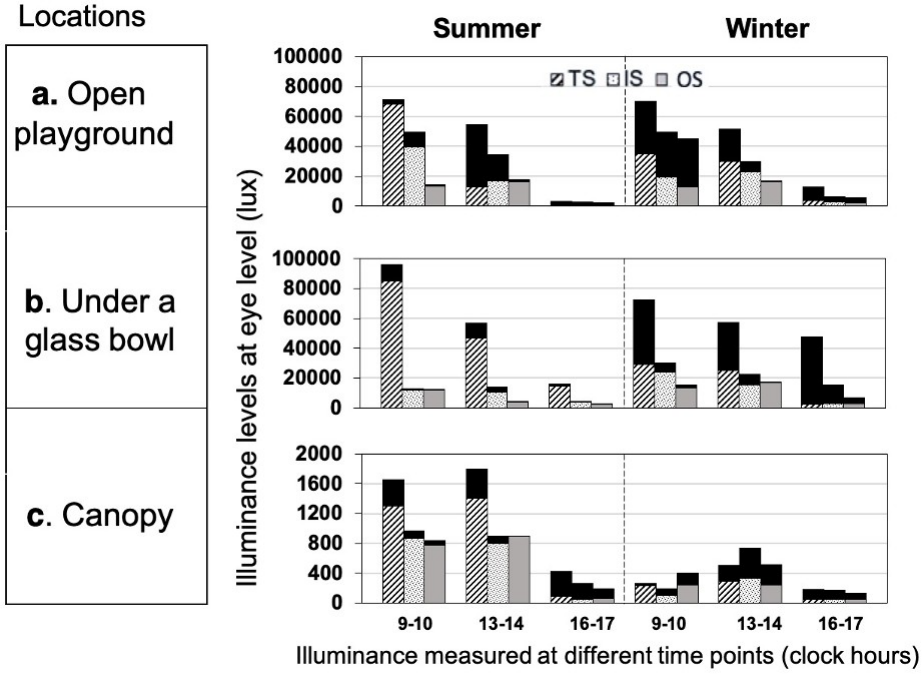
Illuminance levels recorded in three outdoor locations during summer and a winter season. TS, IS, and OS sensor source positions recorded in four different time points of a day (07:00–08:00, 10:00–11:00, 13:00–14:00 and 16:00–17:00 hours). The dark shaded area on the top of each bar represents the difference in illuminance levels obtained on two different days.

Median (IQR) illuminance levels in each of the 3 outdoor locations where the illuminance was determined in summer and winter did not indicate the influence of seasons (illuminance level changing from high to low level or vice-versa) as shown in Fig 5 (summer vs. winter values in “Outdoor playground”: 13600 (2538–33425) lux vs. 16100 (7250–25475) lux; “under a glass bowl”: 11800 (4350–15900) lux vs. 15150 (10600–29700) lux; and “canopy”: 800 (267–900) lux vs. 210 (124–358).

## Discussion

This study described the median (IQR) illuminance levels in 9 different outdoor and 4 indoor locations in various conditions. High illuminance levels were recorded in an “open playground” and “under a translucent artificial shade” irrespective of the various conditions such as time of day, weather, sun protection and type of the day. The median illuminance levels in indoor locations were below 500 lux and were about 8 times lesser than outdoor locations. Outdoor locations such as “under a canopy”, “between three buildings” and “within four buildings” recorded median illuminance levels comparable to that of indoor locations.

Low illuminance levels were recorded in all indoor locations and different conditions with the median illuminance levels <500 lux in the current study, which are in agreement with the values reported previously [8, 19, 21]. Lanca et al. [19] reported the illuminance level measured at an eye level using a mannequin in an open field environment to range from 11,080–18,176 lux and was approximately 100 times brighter than indoor locations (112–156 lux) during cloudy days in Singapore. Besides, the illuminance levels were reported to vary from 5556 lux to 7876 lux under the tree. Wu et al. [8] through a portable light tracker sensor reported illuminance of >100000 lux in an open field, 7480 lux under a tree shade and 7600 lux in a hallway. The maximum and range of illuminance values observed in the current study (“open playground” without sun protection:1120–93500 lux, “under a translucent artificial shade”: 3200–33900 lux) or “under a big tree”: 96–15000 lux) is much higher than that reported in Singapore [19] which could be due to differences in the type of a day (we measured illuminance level on sunny days also while Lanca et al. measured illuminance level only on cloudy days), and sensor-source position (we recorded illuminance in 3 different positions which have sensors facing towards the source, opposite to the source and in an intermediate position in 9 diverse outdoor locations). Another possible reason for higher values in the current study could be the placement of the sensor with respect to location of the eye. We manually placed the sensor in front of the eye whereas Lanca et al. [19] placed the sensor inside the eye of the mannequin limiting exposure of sensor to light from periphery. This explanation can be supported by the findings of Dharani et al. [16] who reported a greater range of illuminance values (278919–30311 lux) using a pendant type light meter instead of a mannequin.

Few outdoor locations such as “canopy”, “between three buildings” and “within four buildings” had low illuminance levels (median: <500 lux), possibly due to the blockage of light reaching the level of eye/sensor by solid structures/thick leaves or shade-like roof. These locations showed illuminance levels >1000 lux only at specific time points and in a certain sensor position. Contrastingly, among the indoor locations, “room with multiple large windows” showed the highest illuminance level, and the overall median (IQR) illuminance level ranged from 350–28500 lux with median illuminance value of 2650 lux. Although a “room with multiple large windows” is considered as an indoor location, the illuminance level exceeded 1000 lux throughout the day except in the evening time (17:00 hours). This could be due to the position of the sun (which was the main source of illuminance) in relation to the room. The windows of this location were facing towards the east direction and during evening time when the sun was present in the west, illuminance level dropped below 1000 lux. The findings suggest that not all locations may be having adequate illuminance for preventing myopia onset and progression, raising the currently employed oversimplified recommendation of time outdoors as anti-myopia strategy. There are a few outdoor locations that show poor illuminance levels and at the same time a few indoor locations show high illuminance that might help in myopia control. In the realistic world and technically, for myopia control based on the illuminance, a few of the outdoor locations may be acting like an indoor and thus the definition of time outdoors for myopia control needs more specifications with regards to time of the day, duration, and the type of locations may be beneficial.

While only 3 locations in this study showed median high illuminance of >10,000 lux, three other outdoor locations namely, “under a porch facing south”, “under a porch facing east” and “under a big tree recorded overall median high illuminance levels >1000 lux, irrespective of the time and weather condition (sunny or cloudy). Previous studies have indicated that illuminance level >1000 lux itself might have beneficial effects in either preventing myopia or delaying the onset of myopia [22, 23]. In addition, Wu et al. [8] based on finding from school-based cluster randomized trial reported that exposure to high illuminance (strong sunlight) may not be necessary for myopia prevention and instead indicated that spending greater time (close to 200 minutes per day) in locations with illuminance levels >1000 lux may be sufficient to slow the progression or prevent myopia. Therefore the potential of any indoor, artificial or semi-natural locations that has illuminance levels >1000 lux cannot be ruled out for myopia control in humans [24].

In the current study, sunlight was a major source of light in all the 9 outdoor locations, and therefore the cardinal position of the sun can play a major role in various time points of a day. The positional impact of the sun at a time can be higher in areas such as artificial structures that can block sun rays. In this study, five out of nine outdoor locations were of such kind, i.e. “under a translucent artificial shade”, “under a porch facing east”, “under a porch facing south”, “between three buildings” and “within four buildings”. The other factor that can play a role in illuminance levels is the geographic location of the country in relation to the equator of the earth (i.e. location of country above or below the equatorial will influence the ambient light levels reaching the surface) [4]. This study was conducted in Hyderabad (capital of state Telangana in India with GPS coordinates: 17° N and 78° E) which is positioned north to the equatorial line and thus the extrapolation/generalizability of results from this study to other regions should be made with caution. The median illuminance levels “under a porch facing east” were lesser by 1.5 times than “under a porch facing south” although both were outdoor locations with similar size and nature of porch. This could be due to the higher exposure of sun rays which is positioned relatively towards the south direction of “under a porch facing south”.

The weather conditions, seasons (cloudy day), time of the day (07:00 hours and 16:00 hours), sensor source position (away from the source), and sun protection with cap or hat although showed a reduction in the illuminance levels, did not shift any location from being high illuminance level category (>1000 lux) to low (<1000 lux), which are in agreement with the previous studies [8, 16, 19]. While the difference in illuminance levels between the two sunny or two cloudy days in outdoors varied by a small margin of 12–13%, a greater variation in illuminance levels between sunny and cloudy day was noted. The illuminance levels in outdoor locations on a sunny day were 50% higher than that on a cloudy day. The outdoor location indicated median low illuminance levels on a cloudy day (800 lux). However, “Open playground” and “under a translucent artificial shade” always showed illuminance level >1000 lux in both sunny and cloudy days irrespective of different measurement conditions.

Given that parents may raise a question that on which time of a day is better to be outside and if the light levels are high in specific time to ensure if myopia can be prevented with time outdoors, we have chosen to record measurements at four-time points which includes closer to timings in before or/and after school hours. The findings of this study indicate that time of the day after 7.00 hours and before 17:00 hours will still have light levels >1000 lux in the southern state of India and children might be benefitted by spending time outdoors before and after school hours. Considering the eye and skin related problems [25] due to direct sun exposure and the finding from the current study indicating lesser variation in illuminance level due to use of any sun protection with hat or cap (illuminance with and without sun protection differed by 1.4 times), it should be noted that spending time in outdoors with sun protection during the day through hat or cap may not interfere with myopia prevention activity based on outdoor light exposure. While the current study did not investigate the influence of sunglasses on illuminance, Lanca et al. [19] indicated that combination of hat and sunglasses in the evening could reduce the light levels at the eye and showed values similar to that of indoors and thus one should choose the outdoor location wisely so that the impact of sun protective gear (hat/cap with sunglasses) on illuminance will be minimal.

Considering the indication for bright classroom model [23] with high illuminance as an anti-myopia strategy, we have also measured the illuminance “under glass-bowl” simulating the outdoor glass room to check how illuminance level will be different to that of outdoor conditions. Zhou et al. [23, 26] developed a bright classroom which measured the median (IQR) illuminance level of 2,540 lux (1,330–4,060 lux). The use of de-polished (light-diffusing) shatterproof clear glass with blinds to the walls could have led to relatively reduced median (IQR) values then that reported here. The intention was to show that illuminance “under a glass bowl” (median (IQR): 13300; 4075–20550; maximum: 80200 lux) will be similar to that of the measurements obtained in an “open playground”. Given that children spend most of their time in a school classroom during the day, modifications to classrooms might be worth to provide better ambient light to prevent myopia. The modified classroom can expose elevated light levels and spectra closer to the outdoor locations. In consideration the cost of building glass classroom in reality [8, 18, 27], the option of an indoor classroom with multiple large windows could be considered to ensure children get exposed to ambient light levels of >1000 lux.

The greatest strength of this study is that we investigated illuminance levels in 9 outdoor and 4 indoor locations which are common/regular places where children are likely to be spending their time. Different conditions (four-time points in a day, sun protection, summer and winter seasons, and sunny and cloudy weather) were included while recording illuminance levels to improve understanding of variation in outdoor and indoor environments with regards to the ambient lighting. Illuminance levels recorded at these locations were at an eye level with three relative sensor positions (lux meter) considering a child might face different directions with respect to light sources while doing daily activities. This study was limited by the following conditions: -i) data of only three outdoor locations were reported during the summer season, while both summer and winter season are equally experienced in India. ii) illuminance level was recorded only in Hyderabad city, which is in the southern part of India, but the geographical spread of India is wide where the weather and season can vary with different states. For example- the Himalayan range in the northern region experience lesser clouds compared to other regions. Likewise, states near the northern border of India may experience more foggy weather and blocks sunlight in the winter season as compared to the southern states. Further investigations are warranted for reporting illuminance levels in different parts of India. The current study cannot make recommendations about locations that can be beneficial for myopia given that this study aimed to document how illuminance varies across different locations and conditions but did not investigate its effect on myopia.

In conclusion, it should be noted that not all outdoor locations may provide adequate light exposure for myopia prevention. Based on the illuminance levels recorded with subject to all conditions, the investigated outdoor locations can be labelled as high illuminance regions that recorded illuminance >1000 lux consistently, moderate illuminance regions simulating semi-outdoor locations where the illuminance level varied between being indoors to outdoors and low illuminance locations that recorded illuminance levels <1000 lux in a majority of environmental conditions. It is worth highlighting that illuminance levels reported in the study did not vary with sun protection, time of the day, weather or seasons and thus children should be encouraged to spend time outdoors with sun protection even in the morning or the evenings. Keeping in view of the variation in illuminance in different locations and other environmental factors, it should be noted that the children and parents need to be wisely provided with more details while recommending time outdoors as anti-myopia strategy which are otherwise oversimplified currently.

## Supporting information

S1 File High-resolution images of nine outdoor and four indoor locations. (DOCX)
