# Supplementary material for: Ambient light level varies with different locations and environmental conditions: Potential to impact myopia
Source: PLoS One. 2021 Jul 7;16(7):e0254027. doi: 10.1371/journal.pone.0254027 (PMC8263252; doi:10.1371/journal.pone.0254027)
Supplement: S1 File — (DOCX) [file pone.0254027.s001.docx]

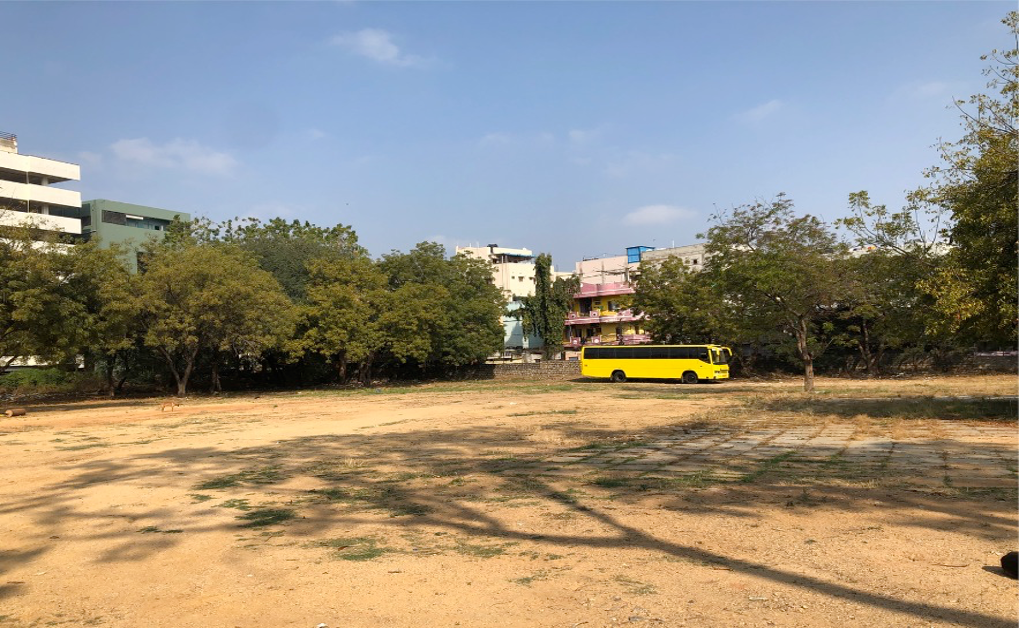
 **Open playground**


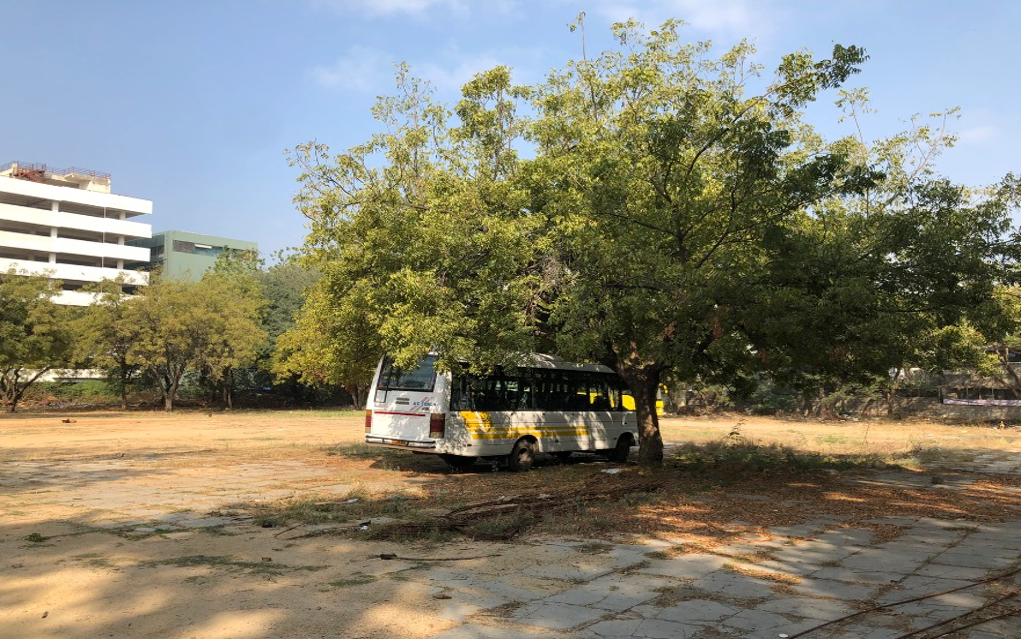


**Under big tree**


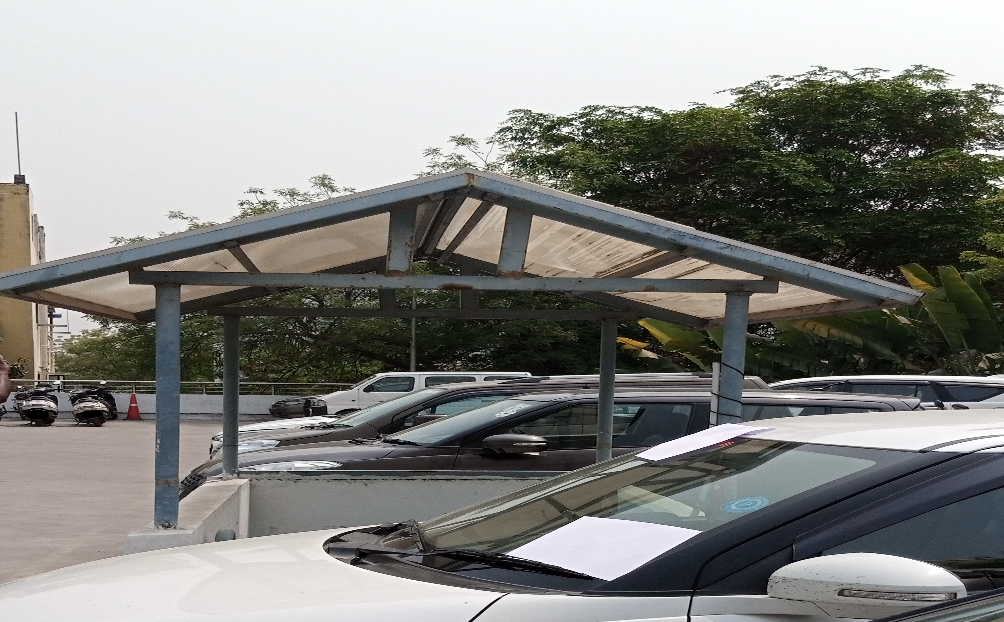


**Under the translucent artificial shade**


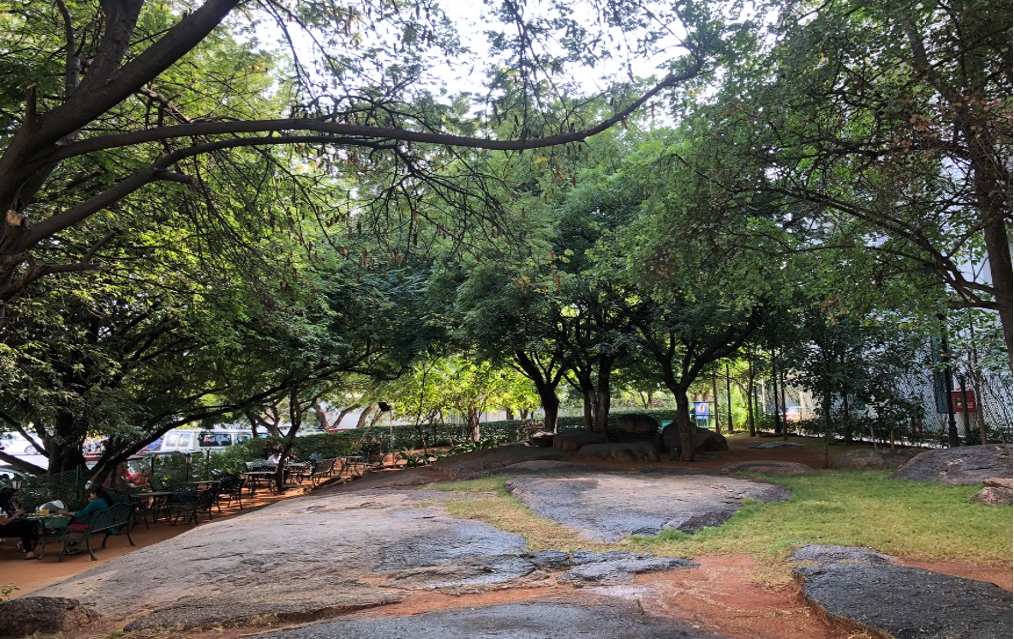
 **Canopy**


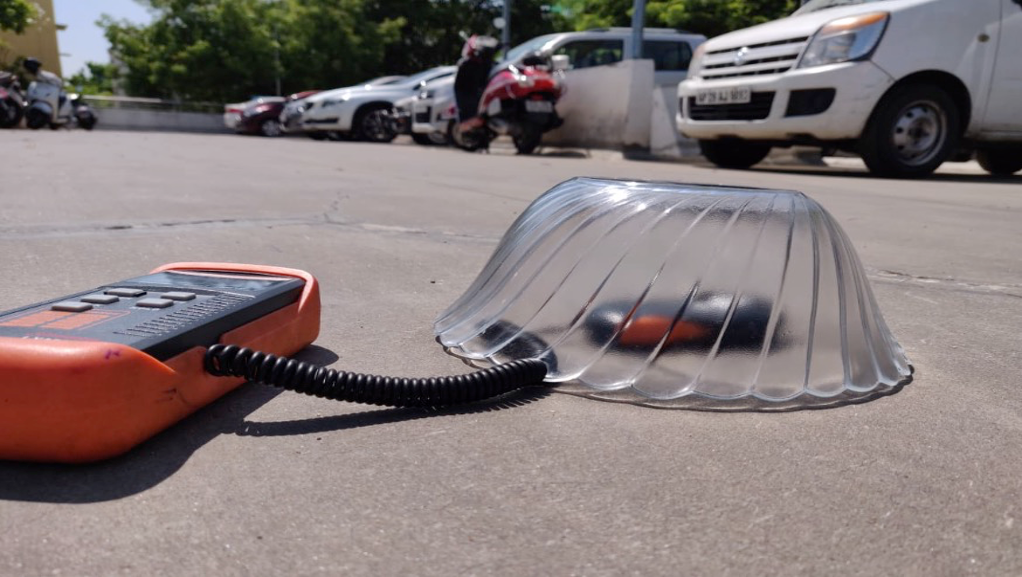
 **Under a glass bowl**


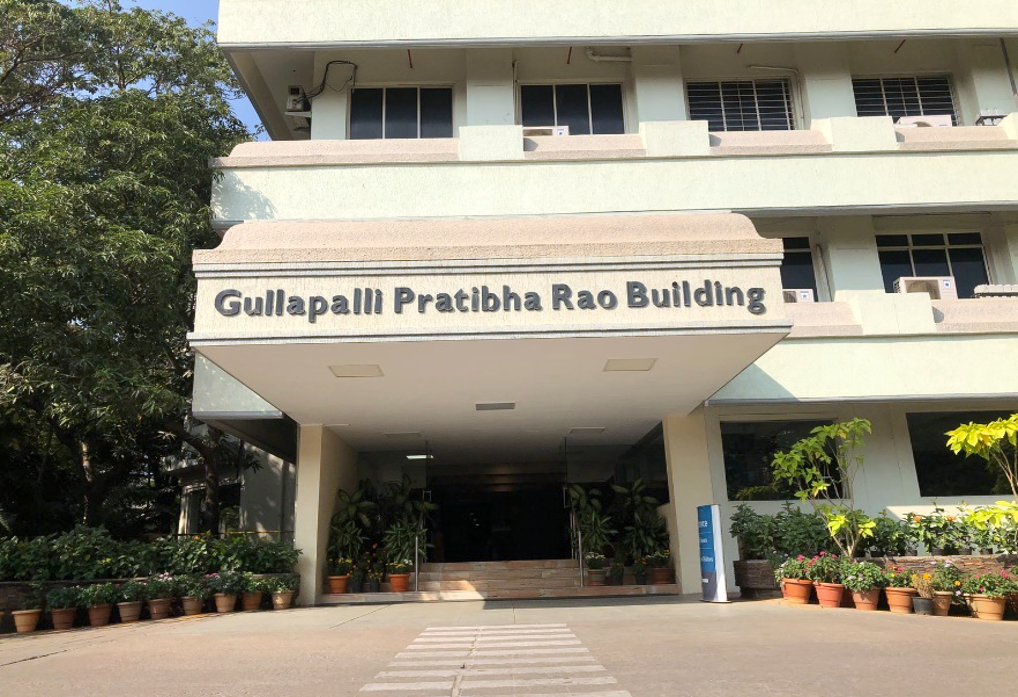


**Under a porch facing east**


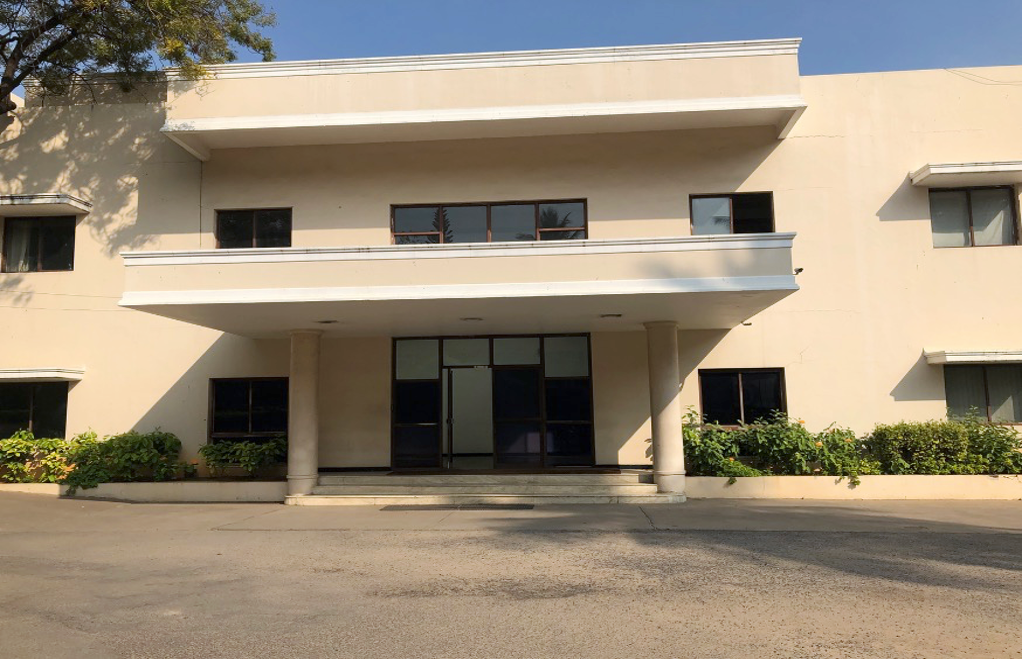


**Under a porch facing south**


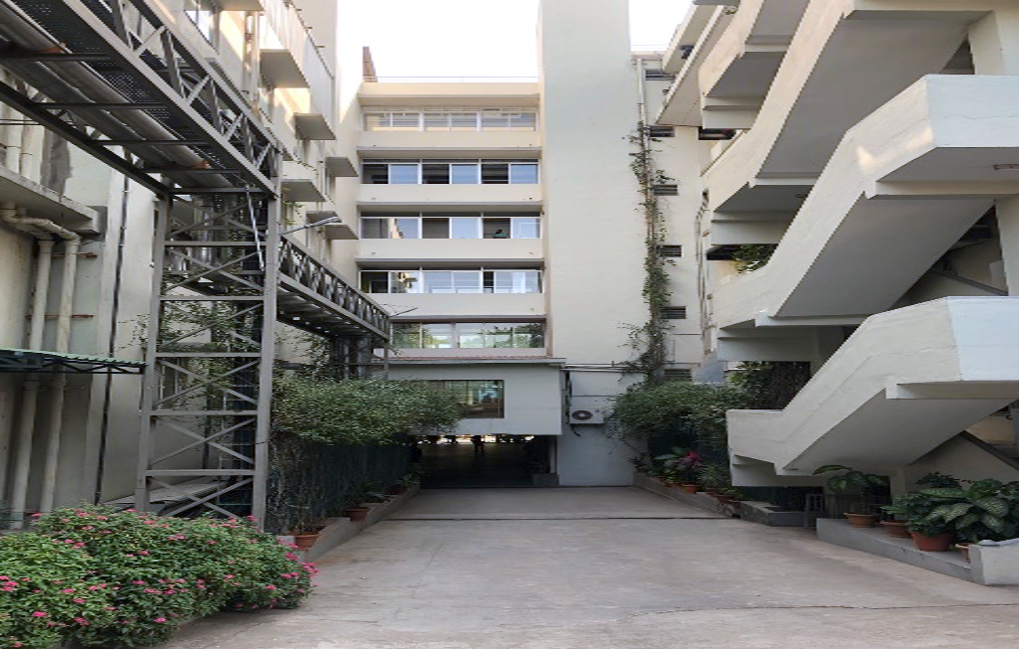


**Between 3 buildings**


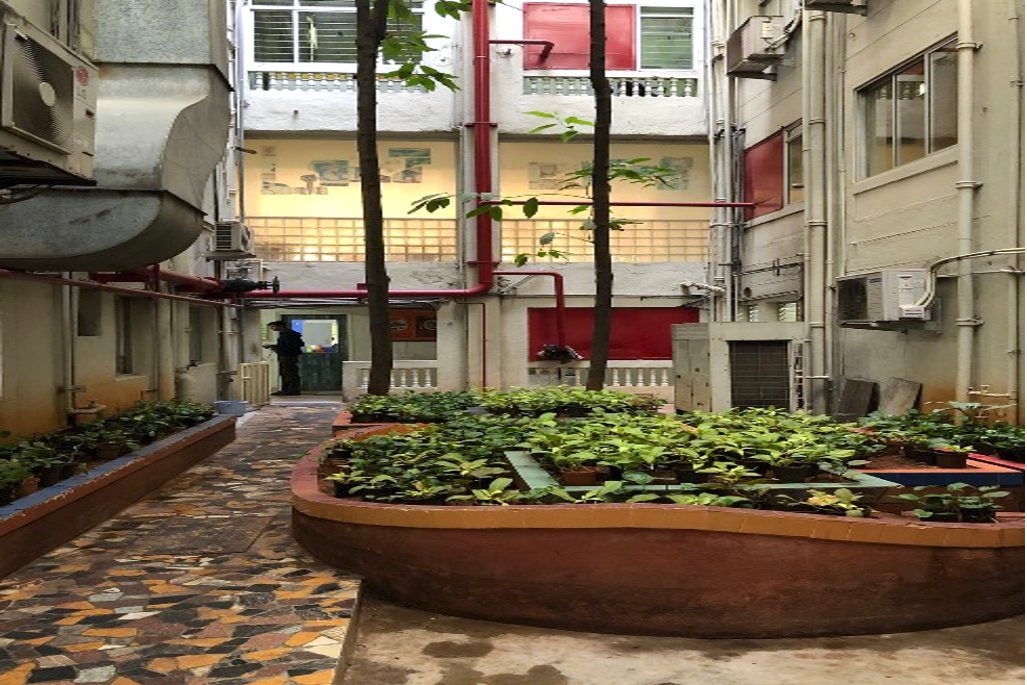


**Within 4 buildings**


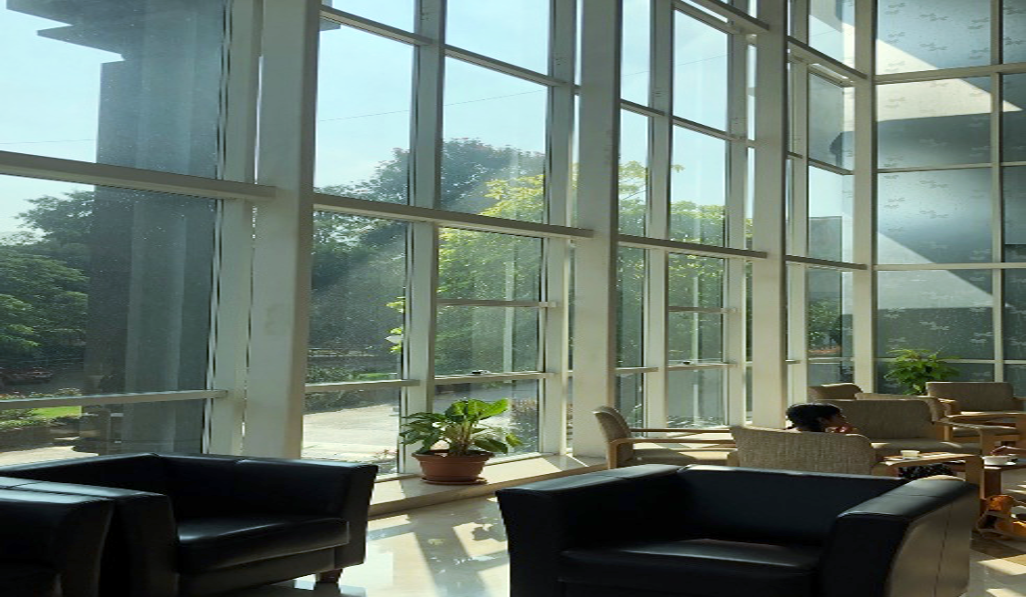


**Room with multiple large windows**


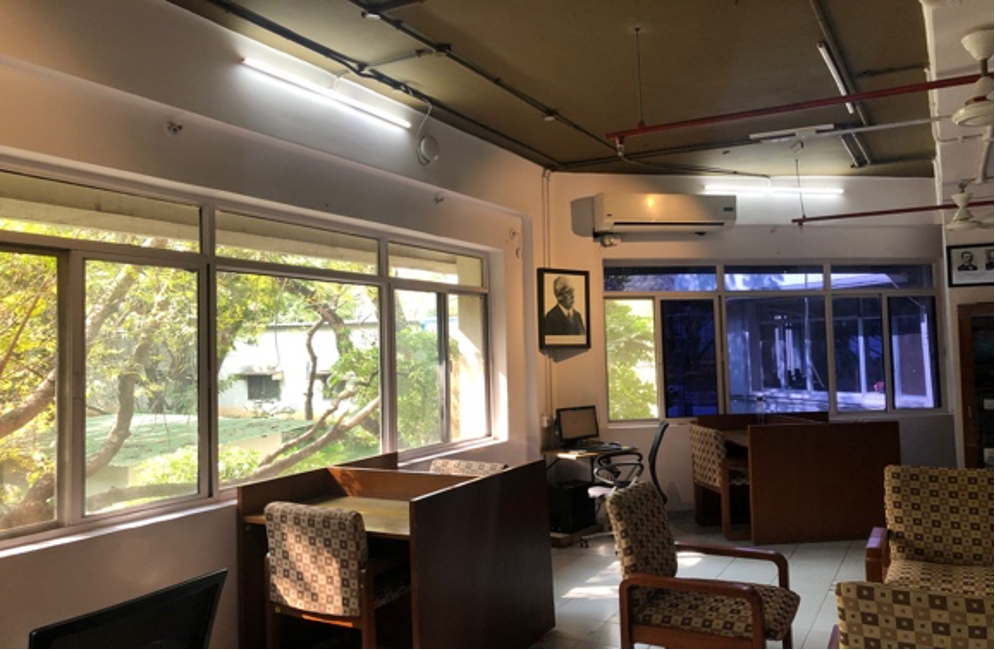


**Room with a combination light source**


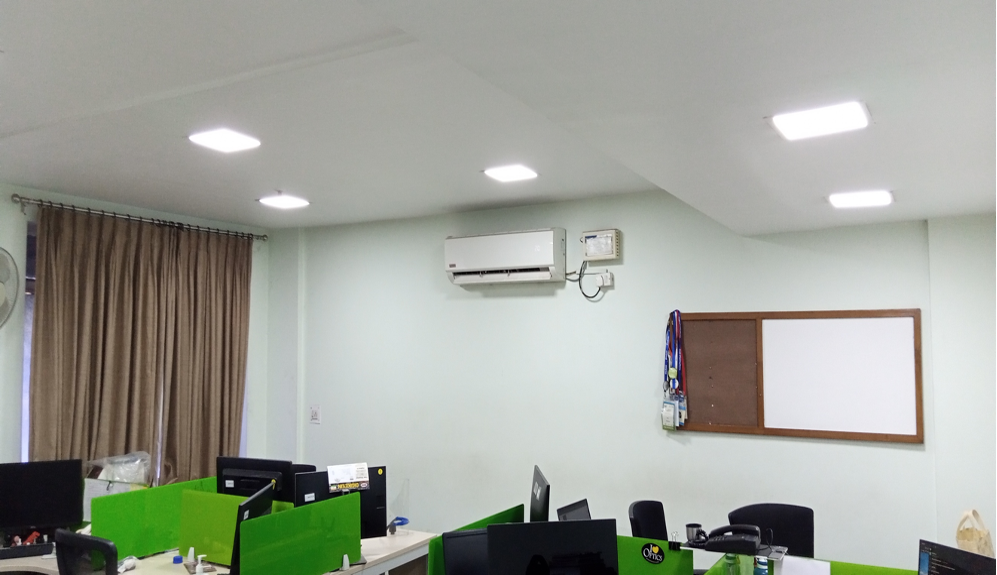


**Room with multiple artificial lights**


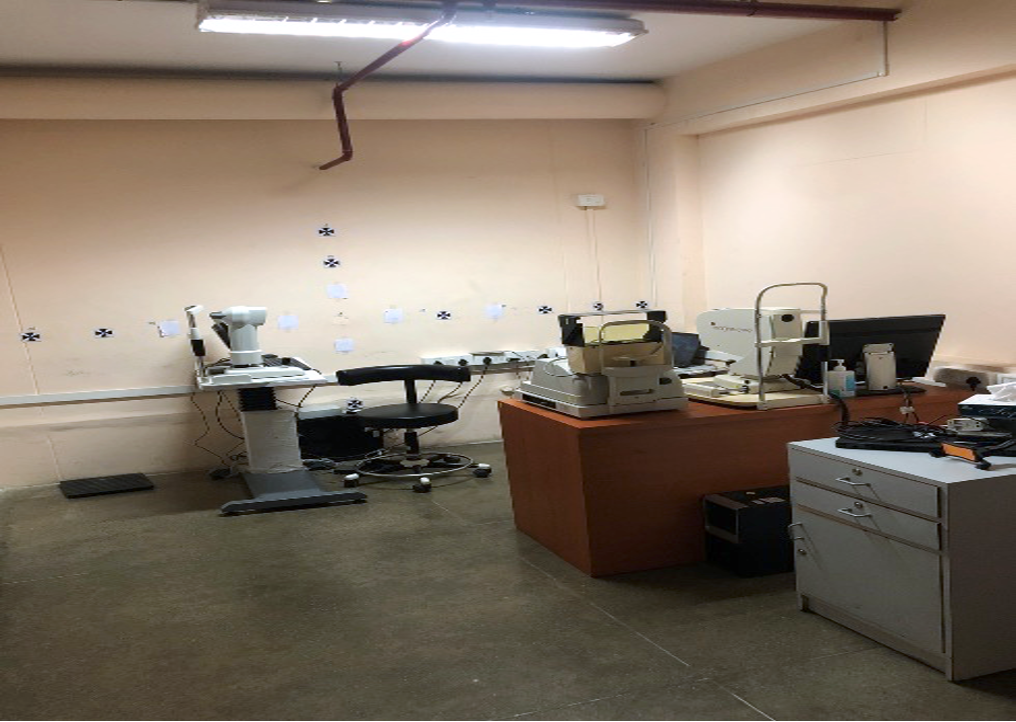


**Room with single artificial light**
